# Temporal evolution of fibroblast responses following salivary gland ductal ligation injury

**DOI:** 10.3389/fdmed.2025.1581376

**Published:** 2025-05-01

**Authors:** Joey R. Tavarez, James Kenney, Sergo Gabunia, Deirdre A. Nelson, Melinda Larsen

**Affiliations:** ^1^ Department of Biological Sciences and The RNA Institute, University at Albany, State University of New York, Albany, NY, United States; ^2^ Molecular, Cellular, Developmental, and Neural Biology Graduate Program, Department of Biological Sciences, University at Albany, State University of New York, Albany, NY, United States

**Keywords:** salivary glands, extracellular matrix (ECM), fibroblasts, injury response, inflammation, single cell RNA-seq

## Abstract

Extracellular matrix remodeling is a natural response to injury but, excessive extracellular matrix accumulation, or fibrosis, is a causative factor in hundreds of diseases that limit organ function, regenerative responses, and can interfere with regenerative therapies. Fibrosis is closely related to inflammation, both of which occur in the salivary glands of patients treated with radiation for head and neck cancers and in patients suffering from autoimmune conditions, such as Sjögren's Disease. Despite the known involvement of fibrosis in disease and the inhibitory effects of fibrosis on tissue regeneration, the mechanisms through which extracellular matrix is elaborated in the salivary gland are poorly understood. Stromal fibroblasts are the primary matrix-producing cells and are known to drive both fibrosis and inflammation. To define the temporal responses of fibroblasts to injury, we induced a temporary obstructive injury though ligation of the primary submandibular and sublingual salivary gland ducts and then performed single-cell RNA sequencing and pathway analysis at timepoints immediately following the injury. Using bioinformatic approaches, we identified three unique fibroblast groups that dynamically respond to the injury. We characterized the changes in matrisomal and inflammatory gene expression over a 7-day time course and identified one group of fibroblasts to be the primary injury-responsive fibrogenic cell type. Understanding how fibroblasts respond at the early and later injury timepoints, along with defining signaling pathways regulated by fibroblasts, could lead to a better understanding of the contribution of fibroblast to acute injury responses to facilitate the development of therapeutics that minimize fibrosis and promote regenerative gland responses in chronic disease states.

## 1 Introduction

The term fibroblast is often used to describe a heterogenous cell population that performs a multitude of roles including extracellular matrix (ECM) elaboration, tissue maintenance and repair, and crosstalk with various cells present in organs (1). Indeed, fibroblasts are the primary cell type that elaborates and remodels the structural and regulatory components of the ECM, known collectively as the matrisome (2). Fibroblasts are known to change their gene expression in response to the environment, and this plasticity is critical to allow fibroblasts to contribute to changing tissue physiology and alter their communication with other cell types. In response to injury or disease, a fibroblast population, referred to as myofibroblasts or fibrogenic fibroblasts, produce the increased and sometimes excessive ECM proteins that make-up fibrosis (3, 4). Fibroblasts are heterogeneous, and recent scRNA-Seq studies highlight the diversity of fibroblast subpopulations present in different organs and in disease states (5–7, 58).

In salivary glands, fibroblasts are essential for organ development and maintaining organ homeostasis in adults, as they exhibit temporal and spatially dynamic deposition and modification of the matrisome. A hallmark of salivary gland damage and disease-associated hyposalivation is the development of a fibrotic response. Importantly, a well-orchestrated fibrotic response is an essential aspect of normal tissue repair and wound healing, while an aberrant and excessive response contributes to progressive organ dysfunction and degeneration (8). This response can be caused by radiation damage, chronic inflammation, or other causes (9–13) and likely contributes to reduced gland function. Recent work has highlighted the heterogeneity in the severity of fibrosis in the submandibular glands of patients with sclerosing sialadenitis (14). Although there have been many recent advances in the development of therapeutic approaches to treat salivary gland hypofunction (15), how to limit salivary gland fibrosis has not been determined. How fibroblast plasticity and function contribute to healing verses pathological salivary gland fibrosis remains unclear, but this knowledge could inform the development of improved therapeutics.

In this study, we examined the temporal changes in fibroblast subpopulation transcriptomes using the mouse submandibular salivary gland ductal ligation injury model, which exhibits a progressive Transforming Growth Factor Beta (TGFβ)-dependent fibrotic response concomitant with gland degeneration (16–19). This injury model was first developed to induce secretory dysfunction in rats that is reversible upon removal of the ligature (20). In prior work, we examined the fibrotic response that occurs 2 weeks after ligation injury in 12 week old female mice (18). Here, we performed single-cell RNA sequencing (scRNA-seq) to identify changes in the fibroblast cell populations immediately following ligation from 1 to 7 days to examine the temporal changes in the construction of the ECM following this acute injury.

## 2 Materials and methods

### 2.1 Animal husbandry

All animal husbandry, surgical procedures, and tissue collection were performed in accordance with protocols approved by the University at Albany, SUNY IACUC committee. Mice were housed in 12-hour light/dark cycle with access to water and dry food. C57BL/6J (JAX #000664) mice were purchased from The Jackson Laboratory. The founders for the B6.129S-Pdgfra^tm1.1(cre/ERT2)Blh^/J (JAX #032770) and B6.Cg-Gt(ROSA)26Sor-tm9(CAG-tdTomato)Hze/J (JAX #007909) mouse colonies were obtained from Jackson Laboratories. To create double transgenic Pdgfrα reporter mice, The *Pdgfrα^CreERT2 +/wt^* (*Pdgfrα^CreERT2^*) males were crossed with *ROSA26^TdTomato +/+^* females to generate *Pdgfrα^CreERT2^; R26^tdT +/wt^* (*Pdgfrα^CreERT2^*; *R26^tdT^*) mice. Mice were assigned a unique identifier between postnatal day 7 and 10 and were genotyped with PCR to detect Cre and tdT in *Pdgfrα^CreERT2^* and *Pdgfrα^CreERT2^R26^tdT^* mice.

### 2.2 Tamoxifen induction

For lineage tracing experiments, *Pdgfrα^CreERT2^*; *R26^tdT^* young female mice were induced at 10–12 weeks old with 3 × 100 µg/g bodyweight tamoxifen (Sigma T5648) injections, as previously described (21). The tamoxifen was first dissolved in 100% ethanol at 55°C then added into corn oil (Sigma C8267). The final concentration was 10% ethanol in corn oil. Injections were performed via i.p. starting 7 days before surgery with three consecutive injections, one every other day.

### 2.3 Salivary gland ductal ligation

We performed pre-operative and operative procedures, as previously described (18). Mice were continuously monitored under anesthesia and post-operatively for pain, distress, and changes in weight for a minimum of 48 h following surgery, in applicable cases. Wharton's and Bartholin's ducts were ligated for 1, 3, or 7 days. As negative controls for the surgical experiments, induced *Pdgfrα^CreERT2^*; *R26^tdT^* mice underwent no surgical manipulations and were euthanized at 12-weeks old. All mice were euthanized at the desired time points under CO_2_ with secondary cervical dislocation.

### 2.4 Single-cell isolation for scRNAseq

For enrichment of stromal cell populations, three mice were used for each timepoint with the mouse's right submandibular and sublingual gland both being harvested together. Excess fat and interstitial tissue were removed. The glands were then transferred to a dish containing 1X phosphate buffered saline (1XPBS), liberase TL Research Grade low Thermolysin (Roche 05401020001) at 0.25 mg/ml, DNase I (Stemcell Technologies 07900) at 15 mg/ml, and dispase (Gibco 17105-041) at 0.53 U/ml and micro dissected for 10 min. The sample was then incubated in a 37°C incubator for 15 min then triturated 100 times. After trituration, the sample was incubated for an additional 5 min in a 37°C incubator and triturated once more. A 15 ml conical tube was placed on ice and the entire sample was transferred to the conical tube and incubated for 10 min. The supernatant was isolated and transferred to a fresh conical tube containing an equal volume of DMEM/F12 (Gibco 11039-021) with 10% fetal bovine serum (Gibco 10082-147) and was centrifuged for 5 min at 150 × *g* 4°C. The supernatant was removed and discarded, and the cell pellet was resuspended in 2 ml of Dynabead isolation buffer composed of sterile-filtered Ca^2+^- and Mg^2+^-free 1X PBS, 0.1% w/v bovine serum albumin, and 2 mM ethylenediaminetetraacetic acid. 5 μg of EpCAM (Invitrogen 14-5791-81) and Ter119 (Invitrogen 14-5921-82) monoclonal antibodies were added to the cell suspension and incubated at 4°C on a rocker set on low speed for 10 min. The cell suspension was washed using isolation buffer and centrifuged at 150 × *g* for 5 min at 4°C. The supernatant was discarded, and the cell pellet was resuspended in 1 ml of isolation buffer.

For depletion of epithelial and red blood cells, 25 μl of sheep anti-rat Dynabeads, which bind to EpCAM- and Ter119 antibody-labeled cells, (Invitrogen 11035) were added. The cell suspension was transferred to a 35 mm dish before incubating at 4°C for 20 min on a rocker set at low speed. The sample was placed on a microcentrifuge tube magnet for 2 min, and the supernatant was collected. One additional epithelial and red blood cell depletion was performed. The sample was then depleted of dead cells using a dead cell removal kit (Miltenyi Biotec 130-090-101), following manufacturer's instructions. Cells were resuspended to a concentration of 1,000 cells/μl and counted using a TC20 automated cell counter (BioRad). Following the manufacturer's protocol for the Chromium Next GEM Single Cell 3' Reagent kits v3.1 (Dual Index) user guide, scRNA-seq libraries were generated.

### 2.5 Single-cell RNA-sequencing analysis

All harvested samples were sequenced on an Illumina NextSeq 2000 at the Center for Functional Genomics - University at Albany, Albany, NY, RRID:SCR018262. Initial processing steps included aligning to the GRCm39 genome with the addition of the tdTomato transcript (22) and generating counts files, which was completed using CellRanger version 8.0.0. Background noise was removed using CellBender (23). Data files were imported using Seurat v5.0.1 in R v4.3.1 (24, 25). Dead or apoptotic cells were removed if >25% of unique molecular identifiers (UMIs) mapped to mitochondrial genes. Any cells with less than 200 or over 9,000 genes were excluded. Potential doublets were detected by DoubletFinder (26) and scDblFinder (27). Droplets that both packages agreed on were removed as doublets. Integration was performed on 12 datasets to create one dataset. The dataset was normalized with SCTransform (28) after integration. Clusters were calculated following the default pipeline (29). All analysis of the integrated dataset was performed using the Seurat package in combination with other R packages. Annotated codes used for analysis are available on GitHub (https://github.com/MLarsenLab).

## 3 Results

### 3.1 Single cell RNA sequencing of ligated submandibular glands

**Figure 1:**
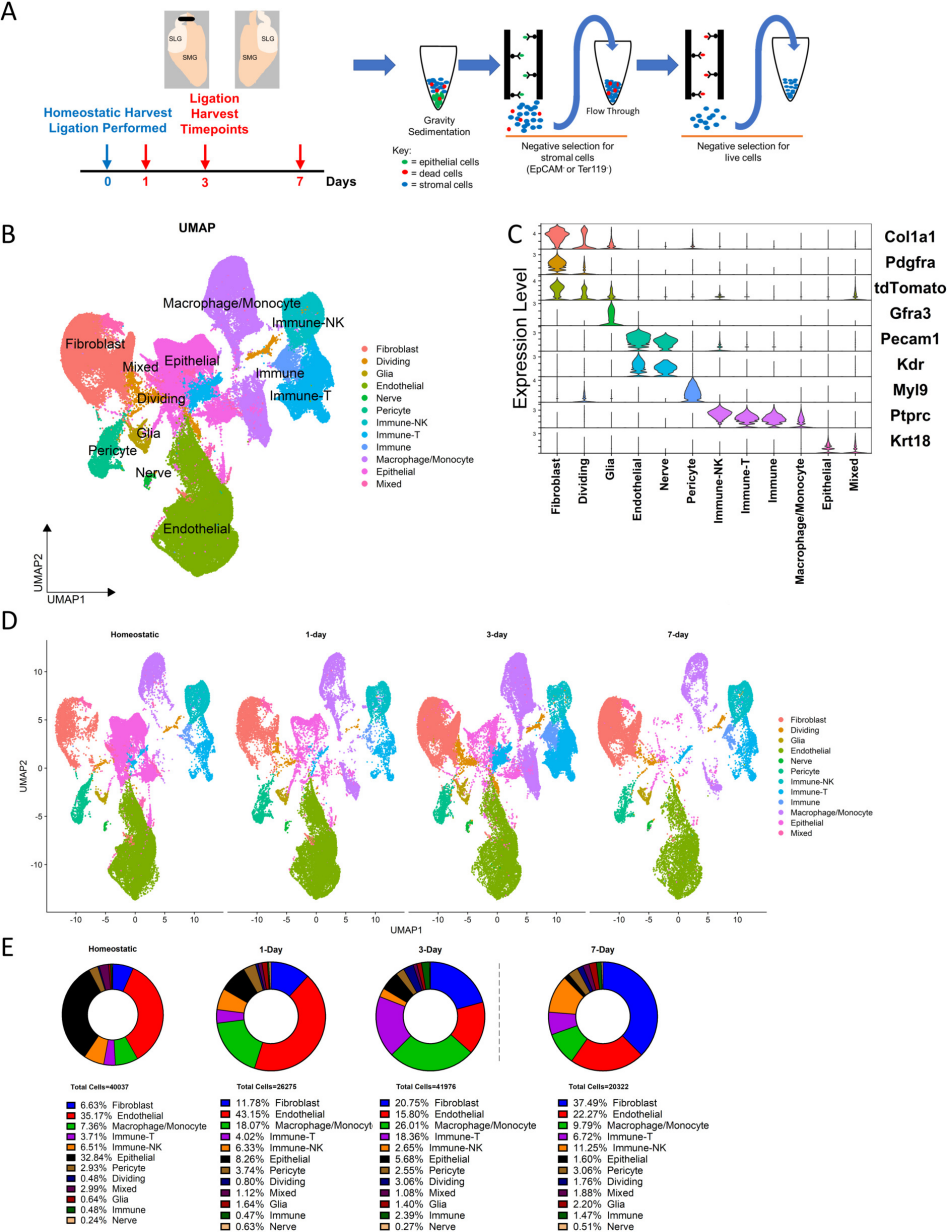
Generation of scRNA-seq dataset in salivary glands 1-, 3-, and 7-days post-ligation. **(A)** Schematic describing the ductal ligation surgery procedure where a clip is placed on ducts exiting the SMG and SLG of young female mice. Tissue was harvested at 4 timepoints (*n* = 3) and subjected to stromal enrichment through gravity sedimentation and MACS after enzymatic dissociation. **(B)** A UMAP displays the cell populations present after processing the datasets and integration. **(C)** A violin plot shows the expression of marker genes used to identify cell populations in the Seurat object. **(D)** A split UMAP shows the respective contributions of each timepoint to the Seurat object. **(E)** Percentages of cells which contribute to cell populations at each timepoint.

**Figure 2:**
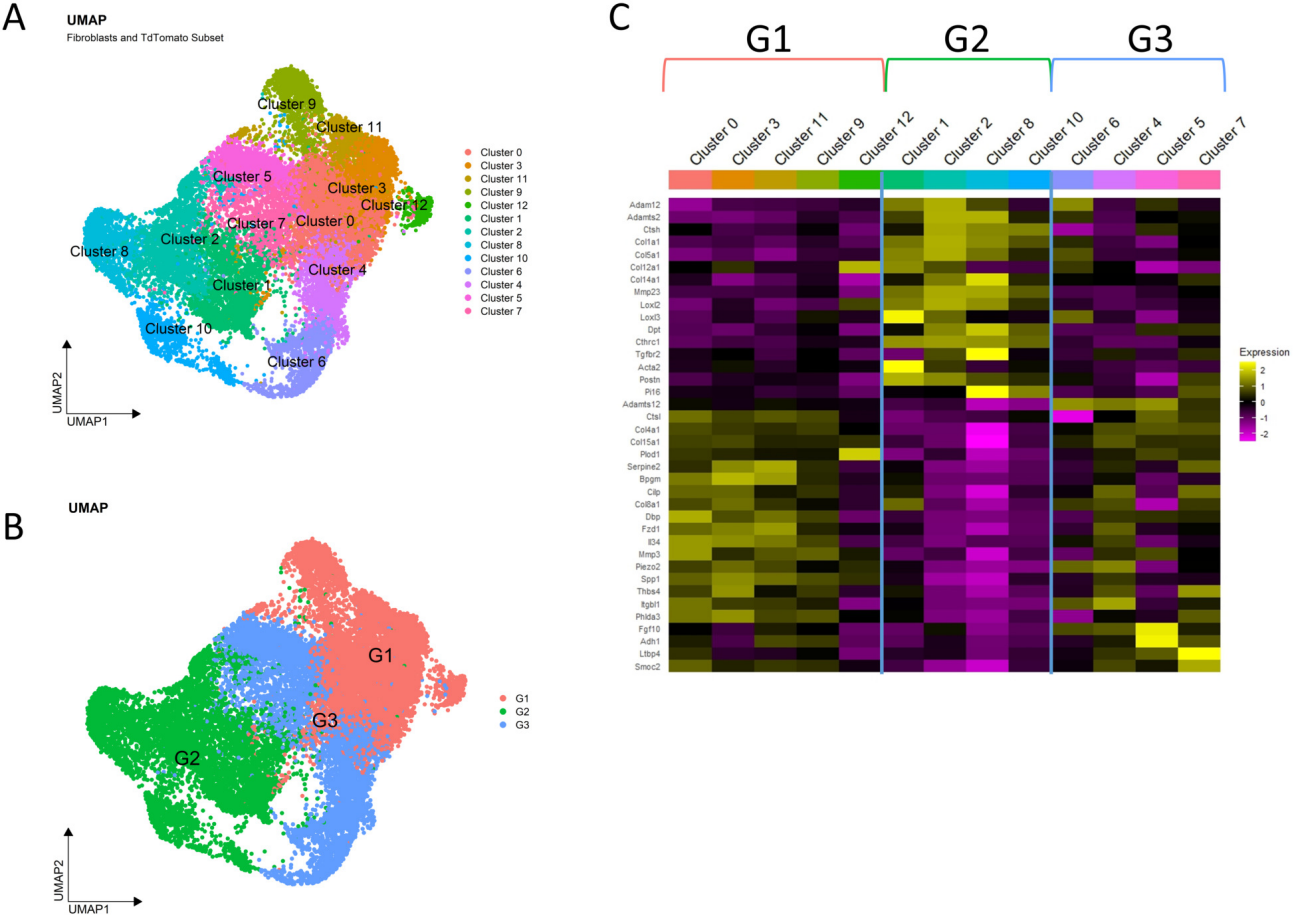
SMG fibroblasts are a heterogeneous population. **(A)** UMAP displays the fibroblast subset obtained by selecting for cells labeled as fibroblasts or expressing tdTomato. **(B)** UMAP shows the 3 groups used to bin clusters that express similar transcriptomes. **(C)** Heatmap shows selected representative genes which highlight the transcriptomic similarities and differences that were used to justify grouping fibroblast subclusters into groups.

To induce a progressive fibrotic response, we performed the ductal ligation reversable injury model to female 10–12-week-old mouse submandibular and sublingual salivary glands by applying a metal clip to Wharton's and Bartholin's ducts, respectively, proximal to the glands (Figure 1A). We used *Pdgfra^CreERT2^*; *R26^tdT^* mice and collected glands from the homeostatic pre-injury state and at timepoints shortly after injury (e.g., 1-, 3- and 7-days post-injury) to reveal early dynamics in the *Pdgfra*-expressing fibroblasts that presumably drive the fibrotic response (Figure 1A). After gland dissociation and enrichment, the stromal cell populations in the tissue were subjected to drop-seq single-cell RNA sequencing. After using Seurat to perform unsupervised clustering, the expected cell clusters were detected in homeostatic glands and ligated glands across all surgical timepoints (Figure 1B,C and Supplementary Figure S1A). Significantly, we detected a progressive increase in the relative contribution of the fibroblast population during the injury time course, that peaked at 7 days. Inflammatory cells, including T cells and macrophages, peaked at 3 days, and NK cells peaked at 7 days (Figure 1D,E). The fibroblasts were then isolated with the subset command, selecting cells that were labeled as fibroblasts by their *Pdgfra* expression or cells that expressed *tdTomato*, creating a new Seurat object. After performing unsupervised clustering, the Seurat object contained 13 distinct subclusters (Figure 2A, and Supplementary Figure S2). Differential gene expression analysis revealed transcriptomic similarities and differences among specific fibroblast subclusters that were then binned into 3 distinct groups based on these similarities (Figure 2B,C).

### 3.2 Characterization of the fibroblast subgroups

**Figure 3:**
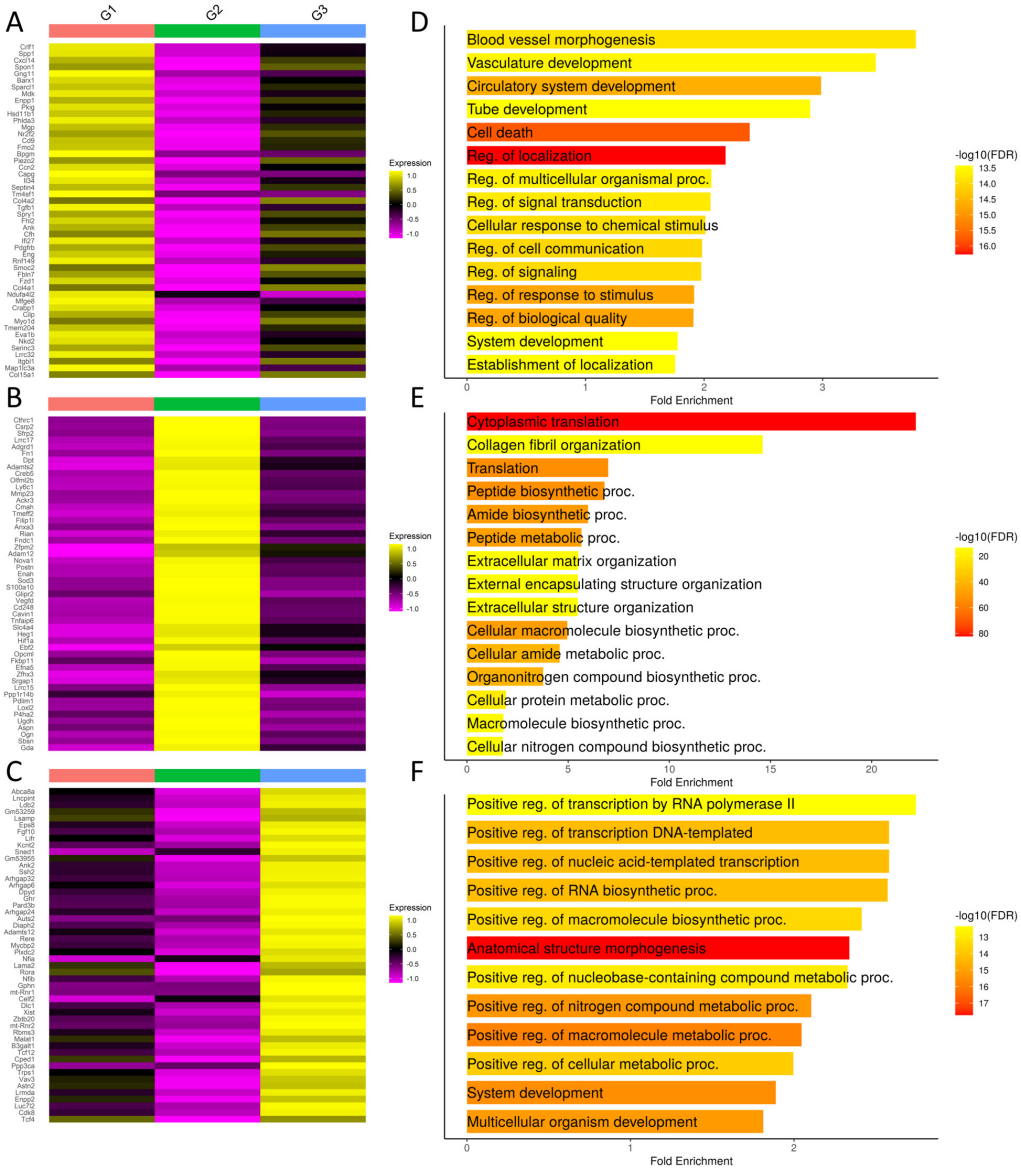
Differential gene analysis in fibroblast groups. **(A–C)** Heatmaps show the top 50 differentially expressed transcripts by each group. Group orders are from top to bottom; Group 1, Group 2, and Group 3. **(D–F)** GO term analysis performed using ShinyGO with the top 500 differentially expressed genes. Order from top bottom is Group 1, Group 2, and Group 3. Refer to Supplementary Tables S1–S3 for complete differentially expressed gene lists.

Heatmaps and GO term analysis of the top 50 differentially expressed genes in each fibroblast group highlight their unique transcriptome profiles that define them as groups and are suggestive of functional differences between the groups (Figure 3, Supplementary Tables S1–S3). Group 1-enriched transcripts exhibit cellular communication and morphogenesis signatures (Figure 3A,D). Cells in this cluster express *Piezo2* and Transforming Growth Factor Beta 1 (*Tgfb1*). Platelet Derived Growth Factor Receptor Beta (*Pdgfrb*) is a profibrotic mediator that is uniquely expressed in this population along with Secreted Phosphoprotein 1 (*Spp1*). Interleukin 34 (*Il34*) is also expressed in this cluster. Together, these data suggest that the group 1 fibroblasts are responsive to mechanical changes in the ECM composition and are both pro-inflammatory and pro-fibrotic.

Group 2-enriched transcripts exhibit an over 20-fold enrichment in cytoplasmic translation and an extracellular matrix signature with enrichments ranging from 5 to 10 fold in ECM-related categories (Figure 3B,D). In this cluster, Collagen Triple Helix Repeat Containing 1 (*Cthrc1*) is expressed. Secreted Frizzled Related Protein 2 (*Sfrp2*) and Dermatopontin (*Dpt*) are also expressed. We see increased expression of *Loxl2* expression which is a member of the lysyl oxidase family proteins that crosslink collagens and elastin to stabilize ECM structure during fibrosis (30, 31). Thus, group 2 fibroblasts are likely to be the primary mediators of ECM deposition and structural modification during the fibrotic response to injury.

Group 3-enriched transcripts exhibit metabolic and synthetic signatures, suggesting that these cells may be critical for directing and maintaining tissue structure and function (Figure 3C,F). Noteworthy genes expressed include Leukemia Inhibitory Factor Receptor (*Lifr*), which has recently been described as a master amplifier of fibrosis in idiopathic pulmonary fibrosis (32). Fibroblast Growth Factor 10 (*Fgf10*), which is a critical mediator of salivary gland development and adult progenitor cell function, are expressed by cells in this cluster (33, 59). These changes are consistent with a primarily a homeostatic function for group 3 fibroblasts. In summary, examination of differential gene expression highlights the likely functional separation of the fibroblast subgroups.

### 3.3 Fibroblast populations change in abundance and transcriptomes over injury time course

**Figure 4:**
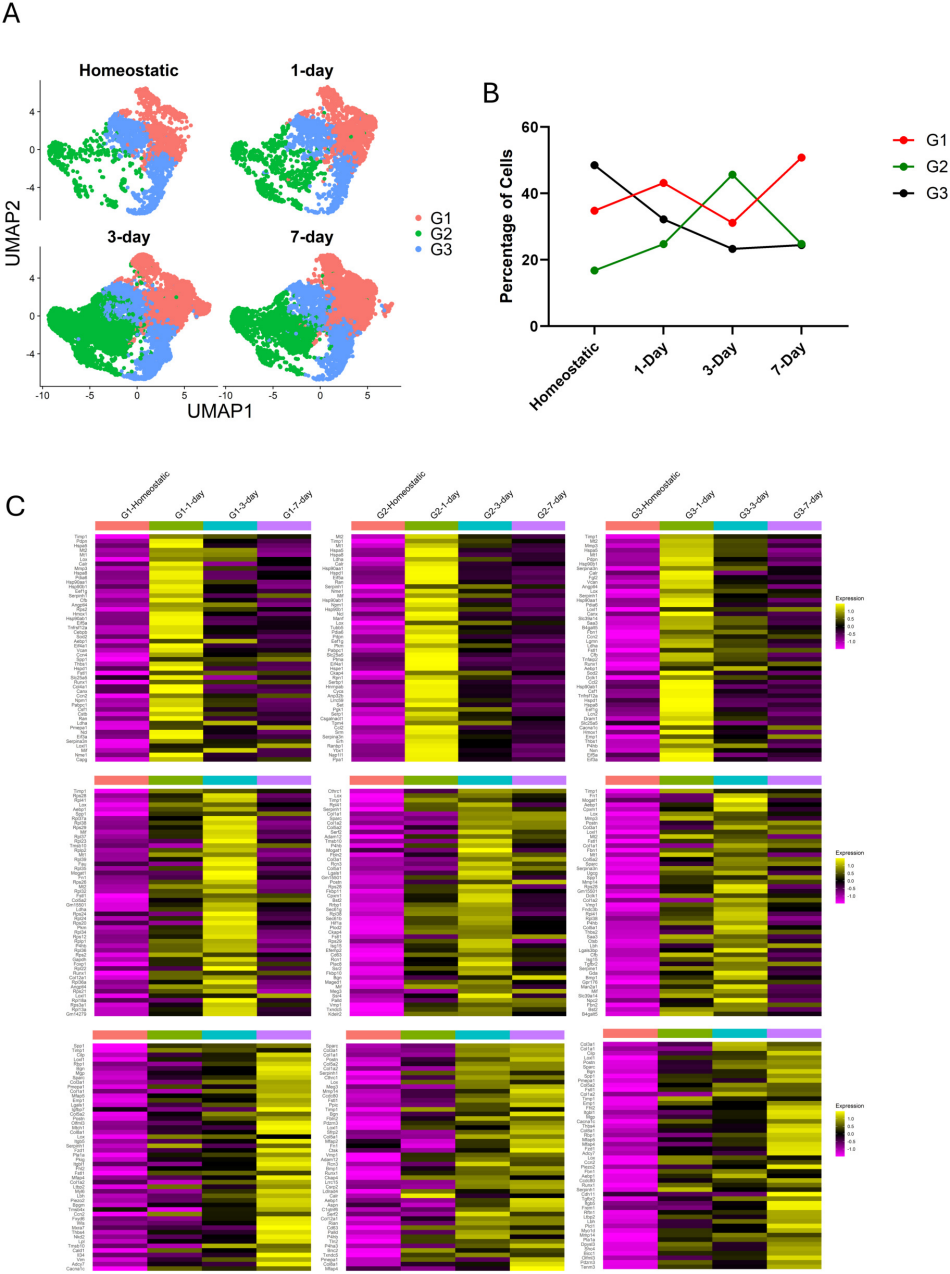
Fibroblast groups exhibit altered transcriptomes at different timepoints. **(A)** UMAPs highlighting the cells from each timepoint and where they are located within the Seurat object. **(B)** Line graph depicts how the populations present in each group change over the course of the injury. **(C)** Heatmaps show the 50 top differentially expressed genes in each group separated by timepoint. All genes shown are upregulated when compared to the homeostatic population in the group. Order from top to bottom is 1-, 3- and 7-days post-ligation. Order from left to right is Group 1, Group 2, Group 3. Refer to Supplementary Table S4 for complete list of differentially expressed genes separated by groups and timepoint.

The abundance of cells in each of the fibroblast subgroups changes over the injury time course (Figure 4A,B). Group 1 fluctuates slightly but is largely stable in abundance over the time course, while group 3 declines in abundance over the time course. Group 2, however, expands dramatically in response to injury. To determine how the gene expression of the fibroblast groups changed in response to injury, we compared the three groups at the three surgical timepoints (e.g., 1, 3, and 7 days post-injury) to the homeostatic timepoint to identify the genes that were differentially regulated.

At day 1 after ligation, all 3 groups exhibit an upregulation of heat shock protein transcripts and cytoplasmic translation, indicative of an acute response to injury (Figure 4C, Supplementary Table S4). There is ubiquitous expression of the chaperone, Calreticulin (*Calr*), in all 1-day ligated fibroblasts. At day 3 after ligation, group 1 has a translation signature and includes selective expression of transcripts such as Fibronectin (*Fn1*) along with *Spp1*, whereas groups 2 and 3 exhibit a more robust fibrogenic signature with large increases in expression of fibrillar ECM genes encoding molecules such as Collagens 1, 3, and 5. We also see the expression of *Postn* and *Cthrc1* which persists at 7-days in group 2. Group 3 includes *Fn1*, *Col1a1*, *Col3a1*, *Col5a1*, *Thbs1*, and Transforming Growth Factor Beta Receptor 2 (*Tgfbr2*). At day 7 after ligation, groups 1 and 3 exhibit a cell migration and morphogenesis signature, suggestive of resolution of acute injury response, while group 2 retains a strong fibrogenic signature. These data indicate that group 2 is the primary fibrogenic population in the injury response.

### 3.4 Dynamics of the inflammatory and matrisome components of the fibrotic injury time course

**Figure 5:**
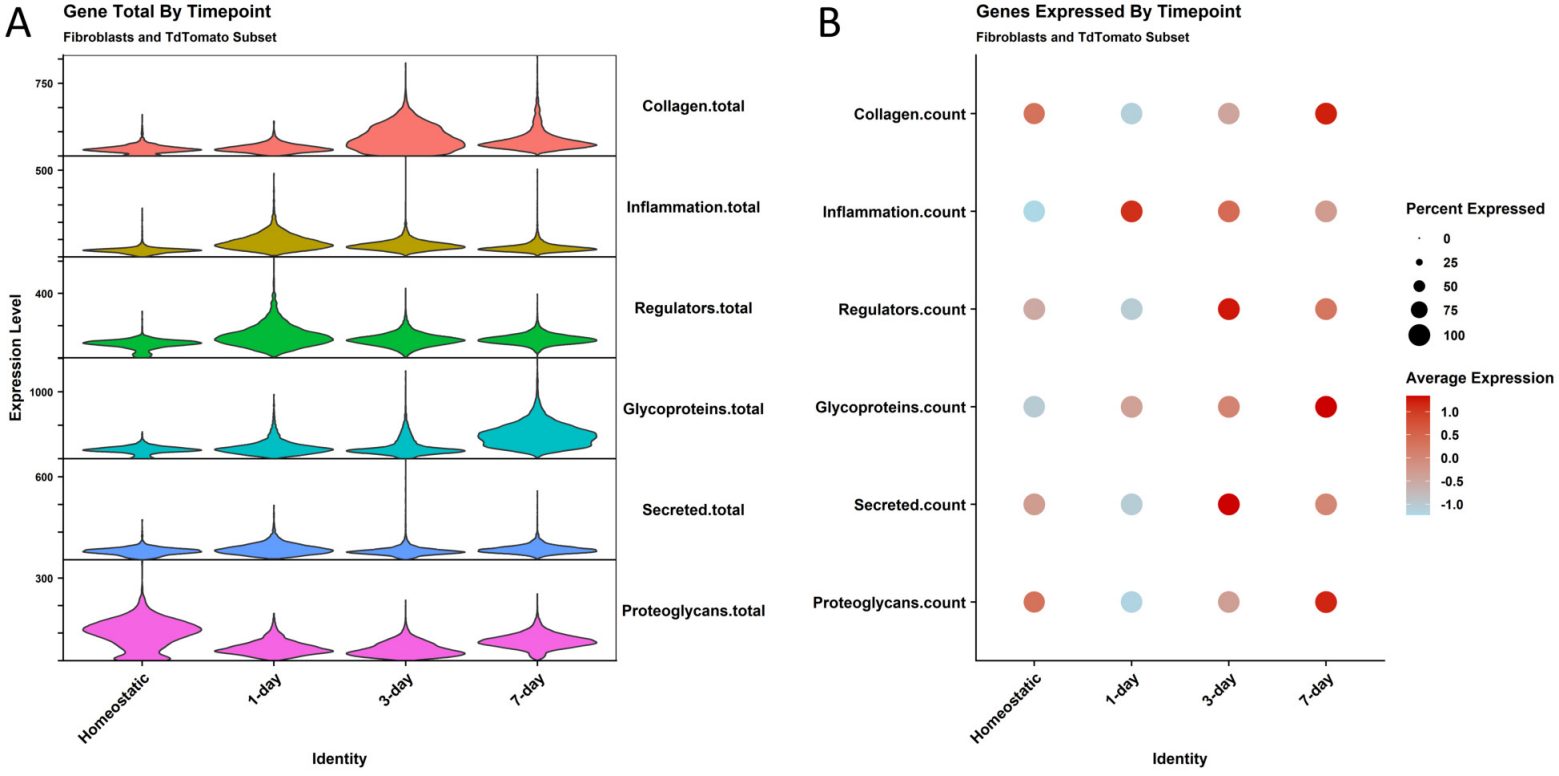
Matrisome gene expression shows changes in abundance and diversity by timepoint. **(A)** A violin plot shows the total transcripts expressed by cells at the different timepoints for gene lists made from the matrisome master list. The gene categories are on the right *Y* axis. Cells are removed from the plot for easier interpretation. **(B)** A dotplot shows the diversity of genes for the same lists.

We then investigated the inflammatory and matrisome transcript dynamics in the fibroblast cells in the fibrotic injury time course. Using Gene Set Enrichment Analysis (GSEA) gene lists curated by Molecular Signatures Database (MSigDB), matrisome components and the hallmark inflammatory response genes (2, 34), we quantified the average number of transcripts that were expressed in these categories over the time course to get a glimpse into the order in which tissue remodeling occurs (Figure 5A). Proteoglycans are expressed at the highest levels at the homeostatic timepoint. 1 day after ligation, we see an increase in inflammatory gene expression and ECM regulators. At 3 days, we see the highest expression of collagen genes by total transcripts, and glycoprotein expression peaks 7 days after ligation. As the diversity of genes expressed in these categories was also of interest, we used a dotplot to examine the number of different genes expressed in the categories (Figure 5B). We see similar patterns to gene abundance with an enriched diversity of inflammatory genes present at 1 day post ligation and secreted factors at 3 days post ligation, but also some differences, such as collagen transcript diversity being the highest 7 days post ligation. ECM regulators also show some difference from total genes expressed with the highest diversity of ECM regulator genes present 3 days after ligation.

We then looked at the individual transcripts related to the matrisome in the time course data to identify contributions of each fibroblast group at each timepoint (Supplementary Table S5). Using this approach, we can see not only which group contributes the most genes to each category, but also at which timepoint these genes are upregulated or downregulated. To normalize for differences in cell numbers, we used transcripts per cell, which is the number of transcripts divided by the number of cells present in each population.

**Figure 6:**
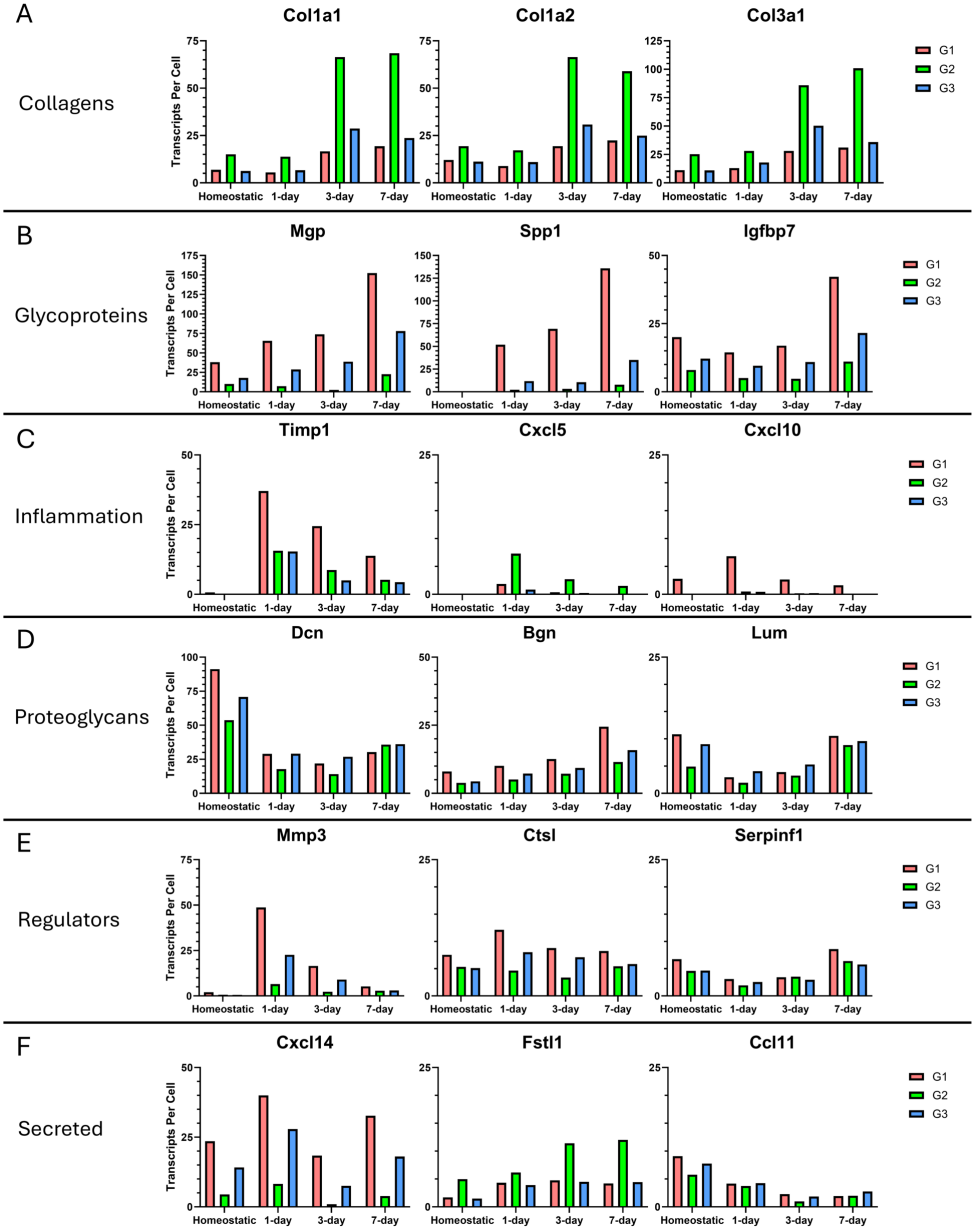
Most abundantly expressed matrisome genes in fibroblast groups 7 days after ligation injury. **(A)** The three most abundantly expressed collagens are shown with the number of transcripts expressed per cell. Each graph is divided into the 4 timepoints. **(B)** Top 3 most expressed glycoproteins. **(C)** Top 3 most expressed inflammation genes. **(D)** Top 3 most expressed proteoglycans. **(E)** Top 3 most expressed ECM regulators. **(F)** Top 3 most expressed secreted ECM proteins. Refer to Supplementary Table S5 for data used to generate graphs.

When we examined the collagen genes, we found that fibrillar collagens (e.g., *Col1a1*, *Col1a2*, and *Col 3a1*) are the main contributors to transcript abundance showing 3-to-4-fold increases relative to the homeostatic population at day 3 and persisting through day 7, with all three genes expressed most abundantly by group 2 cells (Figure 6A). Glycoprotein analysis showed that Matrix Gla Protein (*Mgp*), *Spp1*, and Insulin-like Growth Factor Binding Protein 7 (*Igfbp7*) contribute the most to glycoprotein transcript abundance, and glycoproteins are dominant in group 1 (Figure 6B). Their expression increases steadily beginning at day 1 and continuing to day 7. Compared to the homeostatic state, we detected a 4-fold increase in *Mgp* expression, a 452-fold increase in *Spp1* expression, and a 2-fold increase in *Igfbp7* in group 1 at 7 days post injury. For inflammatory transcripts, Tissue Inhibitor of Metalloproteinases 1 (*Timp1*), and C-X-C Motif Chemokine ligands 5 and 10 (*Cxcl5* and *Cxcl10*) were the top contributors. They were most highly expressed at day 1, and these transcripts were not dominated by a single fibroblast group (Figure 6C). Proteoglycans were the only category to show a decrease in abundance after injury, with Decorin (*Dcn*), Biglycan (*Bgn*), and Lumican (*Lum*) having the highest transcript numbers, and these transcripts were expressed by all fibroblast groups (Figure 6D). Examination of ECM regulators showed that Matrix Metalloproteinase 3 (*Mmp3*), Cathepsin L (*Ctsl*), and Serpin Family F Member 1 (*Serpinf1*) have the highest transcript numbers, with only *Mmp3* being injury-responsive and predominantly expressed by group 1 (Figure 6E). Examination of secreted factors showed that *Cxcl14*, Follistatin-Related Protein 1 (*Fstl1*), and C-C Motif Chemokine 11 (*Ccl11*) contributed the most to the transcript abundance, but these transcripts were not dominated by a single fibroblast group (Figure 6F).

This data reveals a strong fibrogenic injury response in the gland ligation time course, with complex dynamic patterns of these important matrisome genes in the fibroblast subgroups. Group 1 fibroblasts are the main contributors to glycoprotein and enzyme secretion, indicating that this population is the main signaling population present during an injury response. Group 2 fibroblasts are the main contributors of fibrillar proteins making them the main contributors to the fibrogenic phenotype observed. Group 3 fibroblasts may serve primarily a homeostatic function.

## 4 Discussion

The mechanisms regulating a productive, healing fibrotic response and how they differ from pathological fibrosis is topic of considerable current interest for development of improved therapeutics for fibrotic pathologies. Using the reversable ductal ligation injury model in young female mouse salivary glands, we describe a time course of the transcriptomic signatures of this productive fibrotic response. As there are few studies that report transcriptomic changes over time, our time course transcriptomic data will serve as a resource for comparison of the transcriptomic signatures of this productive fibrotic response with resolution of the fibrotic response and with pathological fibrotic responses. Similar to the well-studied wound healing response of skin, we observe an early inflammatory response together with a developing fibrotic response that dominates the fibrotic phase of this reversable injury. We observe a progressive increase in lymphocyte populations such as the macrophage/monocyte and T-cell populations, which peak 3 days after injury. Importantly, the injury-responsive group 2 fibroblasts are the primary fibrotic cells, and these cells show progressive production of ECM proteins from day 3 to day 7.

Different organs and different injuries have distinct fibroblast trajectories and mediators; however, we do detect many universal fibroblast markers (5) expressed by the fibroblasts in our transcriptome dataset, indicating that fibroblasts within salivary glands are similar to other described organs and highlighting the potential for conserved therapeutic approaches. An example of this can be found in group 2 fibroblasts which express Secreted Protein Acidic and Cysteine Rich (*Sparc*), and *Col3a1* which has been reported to be expressed by fibroblasts found in a perivascular niche in multiple inflamed human tissue samples including salivary glands of humans with Sjogren's Disease (35). Notably, group 1 fibroblasts have expression of genes associated with vasculature development and yet group 2 fibroblasts express *Sparc* and *Col3a1* which are associated with perivascular fibroblasts. Perhaps these two fibroblast populations function synergistically to promote vasculature development. Related to Korsunsky et al, fibroblasts associated with T lymphocyte niches that express *Cxcl10* and *Ccl19* were not found in our dataset, which is not surprising, as this is a simple injury model.

Here, we continued previous work which highlighted the transcriptomic differences in salivary gland fibroblasts 14 days after ligation (18). At 14 days after injury, we detected upregulation of similar genes that we here detect increased 7 days after injury. These changes observed in our groups are indicative that the earlier changes persist throughout the injury. Because of the larger numbers of cells in this current study, we can identify distinct fibroblasts subpopulations which seem to have distinct functions. Of note, these different fibroblast subgroups may communicate as ligands and cognate receptors can be found expressed by different fibroblast groups as is the case with *Tgfb1*, which is expressed by group 1, and *Tgfbr2*, which is expressed by group 3. This regulation may enable fibroblasts to dynamically respond to ECM changes allowing secretion of proteins as the need arises.

Our 1-day timepoint sheds light on the initial inflammatory response seen after damage to tissue. Earlier in the injury, we see expression of chemoattractants like *Csf1*, which increase at day 1 but are reduced as the injury progress, we also see widespread expression of heat shock proteins which serve as chaperones, assisting in protein folding but also have functions in activating degradation of aberrant proteins via the endoplasmic reticulum-associated protein degradation pathway (36, 37).

The abundance of fibroblasts allows us to not only see the changes over time, but also the unique subpopulations that are present and the genes that lend to their uniqueness. Genes expressed by group 1 include *Piezo2* which is a mechanosensitive ion channel that responds to matrix rigidity (38), a known hallmark of fibrosis (39, 41) and *Pdgfrb* which mediates PDGF-BB-induced proliferation and migration (42–44). *Cthrc1* expression has been used to identify fibroblasts producing elevated levels of fibrillar ECM proteins (6) which can be seen in group 2 fibroblasts. These same cells express *Sfrp2* and *Dpt* proteins which have been found in fibroblast progenitor populations that can give rise to myofibroblasts (5, 45). These findings point to group 2 fibroblasts serving as the fibrogenic population in this model.

It is interesting to see the changes in expression of ECM proteins at different timepoints after surgical injury. Here we see that not only are genes upregulated in response to injury, as is the case with the glycoproteins, but we see a large decreases in *Dcn*. Dcn is of interest due to its interaction with collagen fibrils and ability to regulate the diameter of collagen fibrils (46–48). It is possible that the decrease in *Dcn* expression leads to the altered collagen organization detected after ductal ligation injury in previous studies (17, 18). Another notable change is in the group 1 fibroblasts where we see a large increase in *Spp1*. Spp1 has been implicated in attraction of lymphocytes that infiltrate injured tissue (49). *Spp1* is also a potent mediator of cell communication that is dysregulated in many diseases and can promote a myofibroblast transition leading to expression of Periostin (*Postn*) (50). Interestingly, *Postn* is expressed by group 2 fibroblasts suggesting possible signaling from group 1 to group 2.

*Spp1* steadily increases and may mediate communication between cells at later stages as the early response cytokines decrease in abundance. *In-vitro* assays performed using *Spp1* knock-down and overexpression showed that *Spp1* is a potent mediator of fibroblast motility and myofibroblast differentiation (40). We also see the expression of other cytokines such as *Tgfb1* and *Il34*. *Il34* is known to promote inflammation via the Colony Stimulating Factor receptor (*Csfr*), suggesting that *Il34* may be a mediator of the inflammation observed in the ductal ligation injury (51). *Tgfb1* is a pleiotropic driver of fibrosis and a potent mediator of tissue repair (52). We will look to see the role that these genes play in a more 3D context in mouse salivary gland organoids where we can add genetically manipulated salivary gland fibroblasts, as in our prior work (53).

We see progressive increases of collagens, such as *Col1a1*, *Col1a2*, *Col3a1*, *Col5a2*, and *Col8a1* starting at day 3, which succeeds the expression of *Spp1* at day 1. Interestingly, Collagen VIII has been found to be expressed in tumor microenvironments and higher levels of expression results in a lower survival for patients (54). We should also note the differences in ratios of fibrillar collagens type I, II, and V as compared to basement membrane collagen type IV. It has been shown that the relative amount of these collagens can regulate cell polarity and function (55). With salivary gland tissue fibrosis, we see a decrease in saliva production, which may be caused by epithelial cell polarity being disrupted in response to a disrupted basement membrane. While the differential gene expression predicts unique functions of fibroblast subclusters, functional studies are required to validate our findings. Investigating the protein levels and disruption of the genes increased in fibroblast groups 1 and 2 will reveal the genes driving tissue repair that may be altered leading to diseased states.

As this acute experimental injury model is reversible, it will be interesting to evaluate the mechanisms involved in fibrosis remediation in future work. The question of whether a fibrotic response in a patient is reversible or irreversible has great clinical importance. Thus, it will be important to compare this reversible response to irreversible fibrotic responses, as that which occurs with partial gland resection in our prior work (56) and with the long-term fibrotic response that occurs following irradiation for head and neck cancers. As fibrosis is said to be responsible for 45% of all deaths in the industrialized world (57), there is a need to recognize fibrotic responses as irreversible or potentially reversible and to develop effective therapeutics to reverse fibrosis in salivary glands and other organs.

Development of effective therapeutics for fibrotic disease has been difficult and often disappointing. Interestingly, a recent report investigating novel therapeutic targets in idiopathic pulmonary fibrosis (IPF) identified Leukemia Inhibitory Factor receptor (LIFR) as an “autocrine master amplifier” of multiple upstream activators of lung fibroblasts (32). TGFβ1, IL-4, and IL-13 stimulation of fibroblasts required the LIF-LIFR axis to evoke a strong fibrogenic effector response in fibroblasts. Thus, in IPF, LIFR drives an autocrine circuit that amplifies and sustains pathogenic activation of IPF fibroblasts. They suggest that targeting a single, downstream master amplifier of fibroblasts, like LIFR, is an attractive alternative therapeutic strategy that can attenuate the profibrotic effects of multiple upstream stimuli. LIFR is expressed primarily in the group 3 fibroblasts in our dataset, which is not the primary ECM-producing group; however, LIF is strongly upregulated in in the group 3 fibroblasts in response to the ligation injury in these young female mice. Communication between the group 3 fibroblasts and the group 2 fibroblasts may be an important component of the fibrotic response. Future studies will be able to investigate whether the dysregulation of the LIF/LIRF signaling pathway is a driver of pathological fibrosis in salivary gland fibrotic diseases and if this is a universal amplification circuit that may be amenable to therapeutic modulation.

## Data Availability

The datasets presented in this study can be found in online repositories. The names of the repository/repositories and accession number(s) can be found in the article/Supplementary Material.

## Supplementary material

Supplementary Figure 1 UMAP shows all integrated replicates. **(A)** A split UMAP shows the 12 replicates that were integrated into the Seurat object. Cell are grouped based on assigned identities.

Supplementary Figure 2 Differentially expressed genes in fibroblast clusters. **(A)** A heatmap shows the top 5 differentially expressed genes in the fibroblast clusters.
