# Supplementary figures and images for: Temporal evolution of fibroblast responses following salivary gland ductal ligation injury
Source: Front Dent Med. 2025 May 1;6:1581376. doi: 10.3389/fdmed.2025.1581376 (PMC12078207; doi:10.3389/fdmed.2025.1581376)

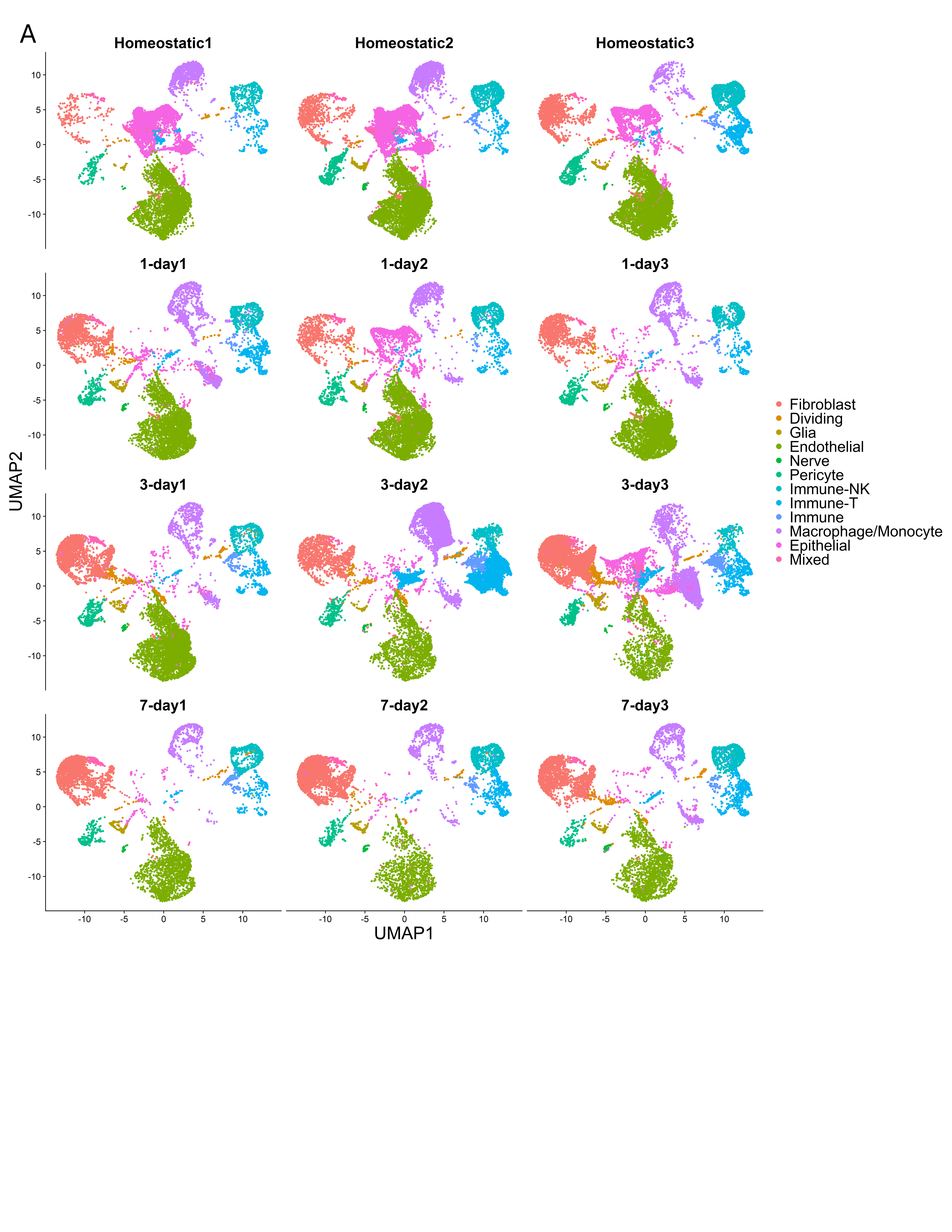

Supplement: Supplementary Figure 1 — UMAP shows all integrated replicates. (A) A split UMAP shows the 12 replicates that were integrated into the Seurat object. Cell are grouped based on assigned identities. [file Image1.tif]

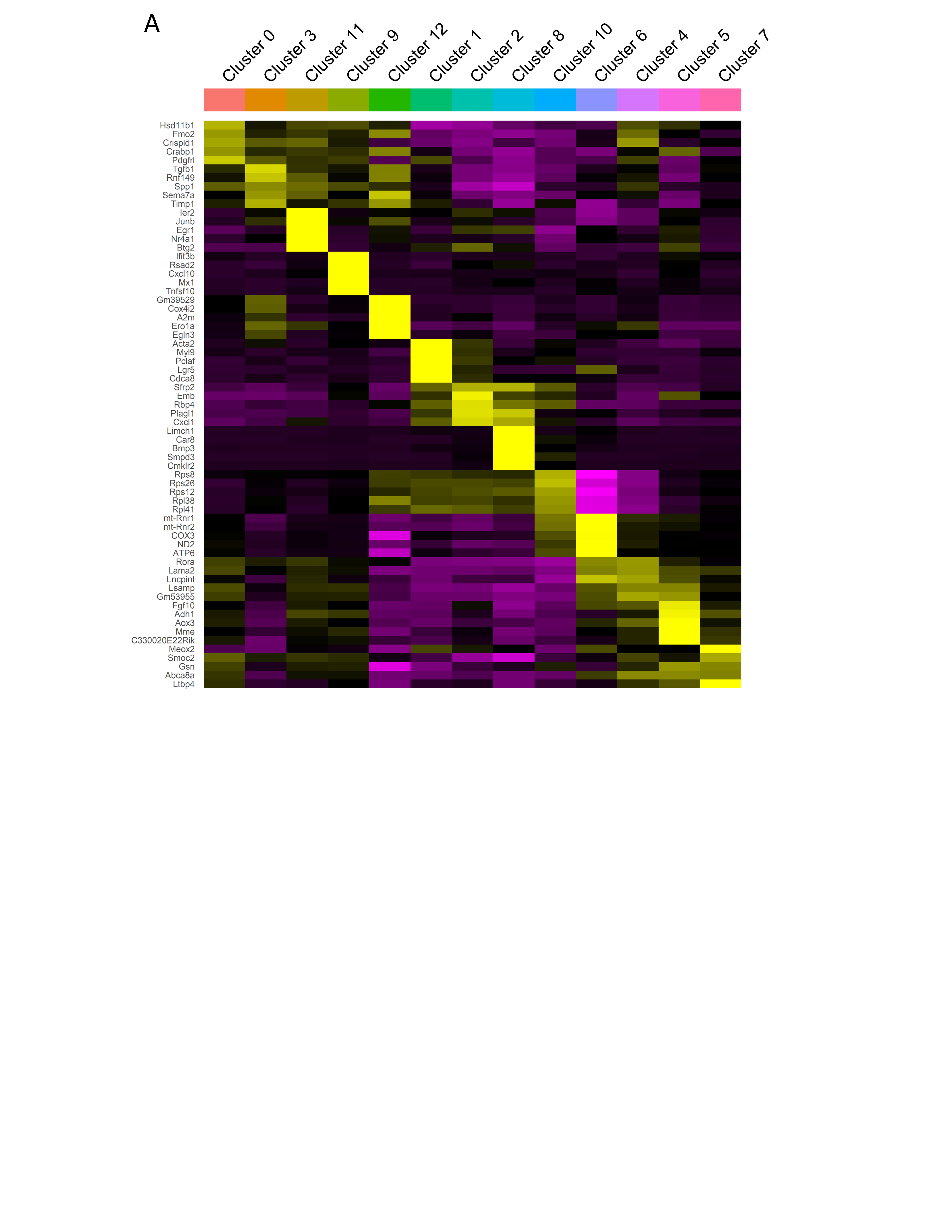

Supplement: Supplementary Figure 2 — Differentially expressed genes in fibroblast clusters. (A) A heatmap shows the top 5 differentially expressed genes in the fibroblast clusters. [file Image2.tif]
